# Feasibility and Preliminary Effects of a Virtual Environment for Adults With Type 2 Diabetes: Pilot Study

**DOI:** 10.2196/resprot.3045

**Published:** 2014-04-08

**Authors:** Constance Johnson, Mark Feinglos, Katherine Pereira, Nancy Hassell, Jim Blascovich, Janet Nicollerat, Henry F Beresford, Janet Levy, Allison Vorderstrasse

**Affiliations:** ^1^Duke UniversityDurham, NCUnited States; ^2^University of California Santa BarbaraSanta Barbara, CAUnited States

**Keywords:** diabetes mellitus, type 2, self-care, user-computer interface, virtual environments software, consumer health information, health communication

## Abstract

**Background:**

Innovative interventions that empower patients in diabetes self-management (DSM) are needed to provide accessible, sustainable, cost-effective patient education and support that surpass current noninteractive interventions. Skills acquired in digital virtual environments (VEs) affect behaviors in the physical world. Some VEs are programmed as real-time three-dimensional representations of various settings via the Internet. For this research, a theoretically grounded VE that facilitates DSM was developed and pilot tested. It offered weekly synchronous DSM education classes, group meetings, and social networking in a community in which participants practiced real world skills such as grocery shopping, exercising, and dining out, allowing for interactive knowledge application. The VE was available 24/7 on the Internet, minimizing access barriers.

**Objective:**

The objective of this study was to evaluate the feasibility and efficacy of participation in a VE for DSM education and support.

**Methods:**

This study utilized a single group, pre-mid-post measure design. At 0, 3, and 6 months, we assessed participants’ perceived VE usability and usefulness, self-efficacy, diabetes self-management behaviors, perceived social support, and diabetes knowledge using validated survey measures; and we recorded metabolic indicators (HbA1c, BP, BMI). Process data were continuously collected in the VE (log-ins, voice recordings, locations visited, objects interacted with, and movement). Data analysis included descriptive statistics, *t* tests to evaluate changes in mediators and outcomes over time, and depiction of utilization and movement data.

**Results:**

We enrolled 20 participants (13/20, 65% white, 7/20, 35% black), with an age range of 39-72 years (mean age, 54 years) and diabetes duration from 3 months to 25 years. At baseline, 95% (18/19) and 79% (15/19) of participants rated usefulness and ease of use as high on validated surveys with no significant changes at 3 or 6 months. Participants logged into the site a mean of 2.5 hours/week over the course of 6 months. High DSM class attendance was reflected by the largest percentage of time spent in the classroom (48.6%). Self-efficacy, social support, and foot care showed significant improvement (*P*<.05). There were improvement trends in clinical outcomes that were clinically meaningful but did not reach statistical significance given the small sample size.

**Conclusions:**

Because relatively little is known about usability, acceptability, and efficacy of health interventions in VEs, this study constitutes an important, innovative first step in exploring the potential of VEs for facilitating DSM. The preliminary data suggest that VEs provide a feasible and useful platform for patients and educators that affects self-management and related mediators. Flexible access to both synchronous and asynchronous diabetes education, skill building activities, and support from a home computer remove barriers to attending clinic-based meetings. This program has potential for improving DSM in an easily disseminated alternative model.

## Introduction

### Overview

“The medium is the message,” a phrase coined over 40 years ago, signifies not only that the content of the message, but also the characteristics of the medium itself affect perception of the message [1]. Digital virtual environments (VE) allow messages to be embedded within realistic sensory scenarios and synchronous conversations among inhabitants affect the perception of the messages. Virtual environments, which promote social and educational interaction via repetition, practice, feedback, and application, may lead to superior learning [2]. Furthermore, virtual environments not only provide the context for the delivery of health messages, but also can potentially bring assistance and support to patients with chronic diseases like type 2 diabetes, to change behaviors needed for effective self-management.

Diabetes affects 23.6 million US adults, most of whom have type 2 diabetes (T2D) [3]. As the prevalence of T2D increases, health care providers and patients face the challenge of effective diabetes management and control to reduce complications and mortality [4-7]. Self-management is integral to the control of T2D, because patients provide 99% of their own care [8,9]. Effective self-management by patients requires informing and aiding patients via ongoing health professional facilitation of monitoring, support, and care, which are notably absent from current health care systems [10-12]. Diabetes self-management (DSM) interventions have demonstrated improvements in diabetes knowledge, self-management behaviors, and metabolic control; however, the interventions and their effects have been relatively short-lived [10,11,13]. The most effective interventions have incorporated interactive, somewhat individualized and frequent interactions among educators, providers, and patients [10]. However, frequent in-person interactions are costly and unsustainable. Ongoing diabetes education and support are necessary in a format that is more sustainable in health care systems [13,14]. Internet interventions, a logical approach to fill this need, have been used with T2D, resulting in increased support [15,16] and knowledge [16]; improvements in glycemic control [16] and self-management behaviors [17]; and more efficient use of primary care services [16] with decreased hospitalizations and emergency department visits [18]. In chronic disease, interactive health communication applications (computer-based information and support) have had a positive impact on knowledge, social support, clinical outcomes, and behavioral outcomes [19]. However, the heterogeneity of the effects of the Internet and technological interventions on metabolic control and self-management, ranging from small to large (and frequently not measured), may be due to a lack of interactivity in these interventions [20] and a drop in utilization of some technologies over time by clinicians and patients [18].

Currently, patients and providers with T2D face barriers to accessing even minimal self-management support or short-term diabetes self-management training (DSMT), including a lack of referral sources for DSMT, fear of losing patients to DSMT if offered outside their clinic, a belief that DSMT was not needed, inability to fit it into their schedule, cost, and transportation [21]. The flexibility of accessing both synchronous and asynchronous diabetes education, skill-building activities, and support via personal computer opens up doors to these aspects of diabetes care for many facing barriers (eg, location, time) to attending traditional clinic based meetings. Beyond these barriers to attending DSMT, VEs provide the potential platform for sustained education and interaction that is necessary throughout the trajectory of this chronic disease.

Digital VEs have the potential to solve the contextual disconnect between the clinic or other education-center based educational programs and the challenges of patients’ daily lives [12] by simulating the community setting, allowing for knowledge application and role playing of self-management behaviors. Instead of being passive observers of computer images, users are active participants in computer-generated three-dimensional worlds. Digital VEs provide users with presence, immersion, and social interaction that can facilitate communication between patients and providers without physical limitations. Virtual environments have the capacity to profoundly change health care from “institution-centric” to “patient-centric” [22], yet further research is needed to determine the feasibility, usability, and outcomes of the technology. Virtual environments have been proposed and explored to address the need for transforming diabetes education and support, and addressing obesity; yet, to our knowledge, formal trials of their efficacy have not yet been conducted.

For this project, a Second Life [23] virtual diabetes community called SLIDES (second life impacts diabetes education & self-management) [24] was built with real-time interactions among adults with T2DM, health care professionals (HCP), and peers. The primary aim was to determine the feasibility and acceptability of an eHealth (a form of health information provided via the Internet [25]) program using a VE platform that provides DSMT and self-management support. A secondary aim was to determine preliminary effects of participation in the VE intervention on self-management and diabetes outcomes (HbA1c levels, blood pressure, and body mass index), as well as psychosocial outcomes or mediators (diabetes knowledge, self-management behaviors, self-efficacy, perceived support, presence, and copresence). The long-range goal is to build a tool for health care providers to not only disseminate information to health care consumers with chronic diseases, but also to promote skill building via interactive simulations and scenarios, provide social support, and offer individual educational consultation.

### Theoretical Framework

Social cognitive theory [26] provides the theoretical framework for the behavioral intervention here, addressing interactive or reciprocal factors in relation to DSMT and support including environmental, personal, and behavioral factors. It was also necessary to incorporate constructs used in the VE literature and theory such as physical and behavioral realism, presence, copresence, and agency [27,28] to address the challenges and opportunities such environments provide compared to face-to-face ones. Details regarding this theoretical framework and how it was operationalized in the VE have been published elsewhere [29]. This integrated theoretical framework allowed us to capitalize on the strengths of successful self-management interventions (eg, the incorporation of social cognitive theory and frequent patient-provider interactions, peer support, feedback, and a multidisciplinary team) and enhance the intervention and participants’ experiences by overcoming the weaknesses of current interventions (eg, a lack of interactivity, accessibility, and sustainability; resource intensiveness) [29].

## Methods

### Overview

This feasibility study involved a one-arm, pre-mid-post measure design. A convenience sample of adult participants with T2D was enrolled in the VE intervention with access to the site for 6 months. Pre-intervention measures at baseline, mid-intervention measures at 3 months, and post-intervention measures at 6 months provided data addressing the primary and secondary aims of the study.

### Recruitment and Study Eligibility

Approval from the Duke University Institutional Review Board was obtained prior to study initiation. Endocrinologists and the study coordinator recruited a convenience sample within the endocrinology clinic at the Duke University Medical Center. Potential participants were identified by endocrinology providers either during scheduled visits or through medical record review. If recipients were interested in learning more or participating, letters were sent to them introducing the study and giving them the study coordinators’ contact information. Eligible participants were those with a diagnosis of T2D who (1) were between 21 and 75 years old, (2) were able to speak and read English, (3) were computer literate (have used a computer for at least 6 months), (4) understood how to use the Internet (have accessed the Internet on at least 6 occasions), (5) had access to a computer with a non-dial-up Internet connection in a private location, (6) were mentally capable of informed consent, (7) were reachable by telephone, (8) had no comorbidities or severe diabetes-related complications that would interfere with study participation or measures (eg, renal failure, stage III hypertension, severe orthopedic conditions or joint replacement scheduled within 6 months, paralysis, bleeding disorders, or cancer), and (9) were able to travel to the clinic for follow-up appointments. Subjects at various stages of T2D with treatment regimens including both oral and injectable medications were included if the above criteria were met.

### Intervention

#### User-Centered Design

The SLIDES community was developed using an iterative usability design, informed by the programmer, researchers, study team, and patients with diabetes [24]. This iterative user-centered design approach was informed by user, task, and environmental analyses [30]. The user analysis determined the characteristics of users such as age, computer literacy, social and cultural issues, and familiarity with Second Life. Hence, we built the environment for adult users who were VE novices, yet who were computer literate and possessed Internet skills. Furthermore, we considered the types of restaurants and types of food in the grocery store that should be included in the SLIDES site based on our user characteristics. The task analysis, which examined the goals of the users and the required tasks to meet these goals, informed the necessary task features of the VE community. For example, we considered that it was important for users to be able to purchase the books that they reviewed within our SLIDES site. Thus, if the user clicked on a book within the bookstore, a website where they could purchase the book directly appeared. In the environmental analysis, we considered types of machines used by the users and the specifications for use of the machines. We stayed within our specifications and did not require the users to purchase any special software to use the site. After we completed the build, we conducted a small-scale usability study with the first 5 participants in the study [31]. Small-scale usability studies are a way to validate the interface design decisions [30,32]. Using a talk-aloud methodology [33], we gave the users 12 most frequently used tasks and functions within the SLIDES environment (such as accessing a menu, navigation, and how to text) and asked them to complete these tasks under the observation of the investigator. The results of this study were used to make some minor changes within the SLIDES site (eg, added menus of popular chain fast-food restaurants, added a sign entitled Medications above the medications in the pharmacy). No major usability problems were found within our launched design.

#### SLIDES Site

The SLIDES site was designed to provide DSMT and support based on social cognitive theory, effective diabetes interventions, and American Diabetes Association and American Association of Diabetes Educator standards for diabetes care and education [34-36]. Classes, resources (grocery item feedback, menu feedback, weblinks, etc), and the infrastructure within Second Life were developed with enhancing patients’ self-management skills and behaviors and interpersonal support in mind. The SLIDES community contained a bookstore with books and websites chosen by diabetes educators with links to purchase them; a grocery store with over 200 interactive grocery items with embedded information on nutritional content, suggested portions and substitutions; a pharmacy with items such as oral care products, glucometers, etc, which could be bought directly online, and information on related medications and over-the-counter applicable medications chosen by our diabetes educators; a clothing store where participants could individualize their avatar; a restaurant featuring the menus of popular chain restaurants with embedded information on each menu item on nutrition, portions, and substitutions; a gym with exercise videos created by exercise physiologists that participants could mimic in their own home; and a community center with a classroom, slide projector, and a forum that allowed participants to cocreate information such as adding information on favorite recipes, restaurants, or personal experiences. This interactive VE incorporated many aspects of social cognitive theory, including cues to action and skill development [37-39]. Role-playing in this diabetes community allowed for immediate feedback and modeling of healthy behaviors. Brief health promotion and disease prevention messages were hung around the site (ie, signs) as cues to action.

**Figure 1:**
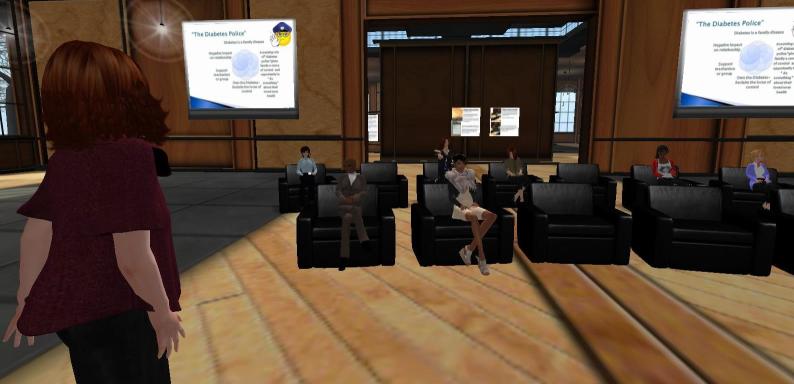
Class session in SLIDES.

Weekly 1-hour classes (using the American Diabetes Association/American Association of Diabetes Educators self-management training curriculum facilitated by nurse practitioners, diabetes educator or health professional guest presenters) were held [35,40] (Figure 1). Classes were offered during daytime and evening hours, allowing participants flexibility for attendance. Twelve classes rotated through the core AADE topics (healthy eating, being active, monitoring, taking medications, problem solving, healthy coping, and reducing risks). Classes allowed for delivery of information by the educators as well as opportunities for participant questions and interactions with one another. In addition, a weekly scheduled discussion/support group was moderated by a nurse practitioner.

Each participant had access to the SLIDES community site in Second Life 24 hours a day, 7 days per week. Access to SLIDES allowed participants to utilize resources and weblinks such as those in the bookstore or grocery store and to interact with other participants at their convenience. Participants were advised to consult their health care provider regarding any medication regimen changes or side effects, symptoms, or health status changes. Medical management remained outside the domain of this intervention.

### Measures

#### Demographics and Internet Experience

Demographic data included age, marital status, ethnicity, race, cohabitation, family history of diabetes, level of education, employment status, and household income range. We also assessed duration of diabetes, diabetes-related medications, attendance at prior diabetes education, and prior attendance at a diabetes support group. Finally, we assessed Internet use in general by hours per week, and then specifically hours per week using social networking sites, emailing, playing games, searching the Internet, and reading newspapers or magazines.

To meet our primary aim of developing a virtual diabetes community (SLIDES) and assessing its feasibility and acceptability, we tracked participation rates (number of log-ins, time spent in SLIDES) and use (locations visited in SLIDES, objects manipulated, voice and text tracking), perceived usefulness, perceived ease of use, and attitudes toward use.

#### Participation Rates and Usage

Process data were measured via the number of log-ins into SLIDES, duration of each log-in, locations visited in the site, and objects touched such as books, food items, etc. Additionally, all verbal and text communication during the duration of the study were recorded continuously (24/7). Process data were analyzed via average number of log-ins per month, average amount of time spent in the site, average amount of time spent in each location within the site, average number of objects touched, and a list of objects touched. Data points such as participant avatar location were collected every 15 seconds, or any time an object was “touched,” to ensure that object interactions were captured in the database.

#### Perceived Usefulness

Perceived usefulness was assessed using a verbal 7-point Likert scale based on work by Davis [41] (Cronbach alpha, 0.98). We averaged participants’ responses to the 6 questions to obtain a perceived usefulness score. Lower scores indicated higher levels of perceived usefulness with responses ranging from 1 (extremely likely) to 7 (extremely unlikely). Questions were modified to fit outcomes related to VEs. The baseline version was modified to reflect participants’ anticipated rating of usefulness of the VE, which could then be compared to the 2 later measures during and after their participation in the VE.

#### Perceived Ease of Use

Perceived ease of use was assessed based on work by Davis [41] (Cronbach alpha, 0.94). The questionnaire included 6 items using 7-point Likert scales that ranged from 1 (extremely likely) to 7 (extremely unlikely). These questions were modified to fit outcomes related to VEs. We averaged the 6 items into a single score of perceived ease of use. Again, the baseline version was modified to reflect participants’ anticipated ease of use of the VE, which could then be compared to the two later measures during and after their participation.

#### Focus Group

The purpose of the focus group was program evaluation. Questions asked during the focus group included: What are your perceptions of the SLIDES site? What did you get out of the SLIDES site? What were positive and negative aspects of the SLIDES program? What changes would you make to the SLIDES program?

To meet our second aim of determining the preliminary effects of participation in the SLIDES intervention, we collected data on metabolic outcomes and potential psychosocial mediating variables as outlined below.

#### Physiological Outcomes

Metabolic control was measured by glycosylated hemoglobin (HbA1c), blood pressure (BP), and body mass index (BMI) obtained from medical records. HbA1c is a routine laboratory measure of metabolic control in clinical practice, obtained at 3-month intervals by diabetes care standards [36] and indicates average glucose levels over the prior 3 months. BP and BMI (calculated from height and weight) are clinical parameters associated with metabolic control and diabetes-related complications (heart disease, stroke) and influenced by self-management.

#### Self-Efficacy

Self-efficacy was assessed using the Diabetes Empowerment Scale-Short Form (DES-SF), an 8-item Likert scale that ranged from 1 (strongly disagree) to 5 (strongly agree), measuring participants’ confidence in their ability to perform DSM behaviors and self-assess satisfaction with diabetes care. The DES-SF was created by choosing the item from the original 28 items with highest item to subscale correlation from each of the original eight conceptual domains (Cronbach alpha, 0.85) [42]. The total scale score was divided by 8 to provide a mean score for each participant.

#### Diabetes Knowledge

Diabetes knowledge was measured using a subset of the Assessment of Diabetes Knowledge [43] instrument. Twenty true/false items were utilized from this 114-item instrument, with representative items selected from the subcategories of treatment, sick days, hypoglycemia, effects of physical activity, reducing complication risks, smoking/alcohol effects, foot care, and diet. This scale was scored based on percentage of correct answers to the 20 items for each participant.

#### Self-Management Behaviors

The 11-item Summary of Diabetes Self-Care Activities [44] assessed the frequency of health behaviors over the previous week (number of days), including diet, exercise, blood sugar testing, foot care, and smoking. The instrument has acceptable internal consistency (mean, 0.47), and moderate test-retest correlations (mean *r*, 0.40) [44]. Responses were scored according to the authors’ instructions, creating a mean score for each of the subscales of diet, exercise, blood sugar testing, and foot care [44].

#### Perceived Support for Diabetes Management

Perceived support for diabetes management was assessed using a 12-item survey with Likert scale items ranging from 1 (strongly disagree) to 7 (strongly agree). This diabetes support scale was developed specifically for assessment of social support in a diabetes Internet intervention. It has demonstrated internal consistency reliability of 0.90-0.93, sensitivity to intervention effects, and construct validity when compared to the Interpersonal Support Evaluation List and Chronic Illness Support Survey (*r*, 0.26-0.45) [15]. Each total score was divided by 12 to calculate a mean score with higher scores indicating more perceived support.

#### Presence

Presence (sense of actually being in the site) evaluation was based on the work of Witmer and Singer [45] (Cronbach alpha, 0.81). The original questionnaire included 32 items; however, we removed 5 items that were not applicable to this environment; 2 questions relating to the haptic subscale (tactile feedback), which is not available within this VE, and 3 questions regarding gaming experiences that were not related to any subscales. The subscales measured involved/control, natural, auditory, resolution, and interface quality. The involved/control subscale focuses on the reaction of the VE to user actions, users’ perception of their control of actions in the VE, the degree of engagement of the visual aspects of the VE, and how engaged the participant felt [45]. Natural subscales assessed the perception of natural movement, the naturalness of the environment, and the participant’s perceptions of natural interactions [45]. The auditory subscale addressed identifying and localizing sounds, and resolution addressed how well the participants could examine objects. Finally, the interface quality subscale assessed whether devices associated with the interaction in the environment interfered with the performance of tasks [45]. The presence questionnaire contained a 7-point scale with anchors based on the question stem from 1 (not) to 7 (very). We averaged each subscale, but do not provide an overall presence score because we eliminated 5 questions. However, each of the subscales should provide some measure of presence.

#### Copresence

Copresence (sense that others are actually present in the environment) [46] was measured based on the work of Bailenson, Swinth, and Hoyt [47] (Cronbach alpha for subscales, 0.71-0.72). In addition to the 3-item scale measuring copresence, we measured embarrassment (3 items) and likability (4 items), both of which help to define copresence by showing social responses people have to others, such as liking others and willingness to perform embarrassing acts in front of others. All of the items were measured on a 7-point Likert scale from 1 (strongly disagree) to 7 (strongly agree). The means were obtained for copresence, embarrassment, and likability, respectively. Greater scores indicated higher levels of copresence, embarrassment, and likability. According to Bailenson et al [47], all three should be correlated: copresence and likability should be positively correlated and copresence/likability and embarrassment negatively correlated.

### Study Procedures

During the baseline participant visit in a private room at our study office, the study coordinator obtained signed informed consent and administered surveys to assess demographic factors and psychosocial mediators as described above. The most recent height and weight, BMI, BP, and HbA1c were obtained from the participant’s medical record by the study coordinator. During the baseline visit, subjects were oriented to the SLIDES site in Second Life and each subject’s avatar was developed by the study staff with the participant’s input and approval. Although participants could keep their individualized avatar indefinitely, they could only access the SLIDES site during the study period. Participants were given headphones with a microphone for home use to allow for synchronous voice communication. Online tutorials that were developed in Second Life helped to facilitate participant learning and integration into the VE. Written instructions for accessing the site were given to each participant along with their username and password during the initial, baseline visit. The initial baseline orientation with the study coordinator was approximately 60-90 minutes long. Most participants required at least 1 follow-up phone conversation with the coordinator to ensure that the application was set up and that they were able to use the basic functions on their home computer, which took about 30 minutes. Although periodic technical challenges required follow-up support, most were either related to Second Life server issues beyond our control or limitations of participant computers (processing capabilities, etc).

Individuals were told that they could visit the SLIDES site as frequently as they wanted, but were instructed to sign in to the SLIDES intervention at least twice a week for the first 4 weeks, after which they could log in to the SLIDES site at any time for the remaining 5 months. The rationale for the initial required log-ins were to encourage use and increase familiarity with the site, resources in the site, and to meet others and attend weekly diabetes education meetings. This was only “required” and participants received reminders from the study coordinator to log in if they did not meet this requirement for the first month so that their use of the site after that point could be observed for the remainder of the study as a part of feasibility testing. Three days after the baseline appointment, participants were telephoned to address any questions about Second Life or study procedures. A re-orientation was provided to those who continued to have difficulty with how to use the site, which was necessary for approximately 4 participants.

Weight, BP, and HbA1c at 3 and 6 months were obtained from medical records (with a 2-week window before or after the study follow-up time point). Subjects were sent a link via email to the follow-up surveys (as outlined above), which were administered online at 3 and 6 months through REDCap [48]. In the final week of the 6-month intervention period, all participants (including those who had withdrawn) were invited to attend an online focus group in the SLIDES site with the primary investigator and coinvestigator to discuss perceptions of the experience, positive and negative aspects of the intervention, technical difficulties, and suggested changes. Subjects who completed all visits and measures received $50 as compensation for their time, or prorated compensation depending on time spent in the study.

### Statistical Analysis

All data, including the process data recorded in Second Life, were stored on a secure research server at the Duke University Office of Instructional Technology. Statistical data analysis was conducted in SAS software version 9.3 [49]. Data to test feasibility and acceptability of this eHealth intervention (primary aim) were descriptive and qualitative. Descriptive statistics were used to analyze the survey data on perceived usability and usefulness of the platform at 3 months and 6 months of study participation. In terms of the process data collected in the SLIDES site, descriptive statistics were used to provide frequencies, means, and standard deviations of the number of log-ins, number of minutes spent logged into the site, number and type of objects touched, and locations visited within the site. Heat mapping data were analyzed in R [50] by computer scientists to show locations visited. The focus group qualitative data were analyzed using content analysis, which is a data reduction technique that examines verbal data for recurring themes. Codes and themes were identified by the researchers based on participant discussion and responses. These focus group members’ responses were categorized into four categories: expectations, positives, problems, and suggestions. These four categories were then coded into themes according to the constructs in our theoretical framework [29]. The text segments were analyzed for frequencies of themes. A second researcher reviewed the content of the focus groups for rigor of the findings.

Addressing our secondary aim, the preliminary effects of our VE program on measures of metabolic control (HbA1c levels, BP, and BMI) and potential psychosocial mediating variables (perceived support, self-efficacy, diabetes knowledge, diabetes self-management behaviors, presence, and copresence) were assessed. Means and standard deviations were used to describe these parameters at baseline. Paired *t* tests were used to assess pre-mid-post intervention differences in the study dependent variables (two-tailed tests, α<.05). Given that this was a small pilot sample, with 61 missing follow-up data points out of 342 data points (18%), we did not attempt more sophisticated models accounting for missing data. In a larger trial and sample, we would have accounted for it in this manner.

## Results

### Demographic and Internet Experience

**Figure 2:**
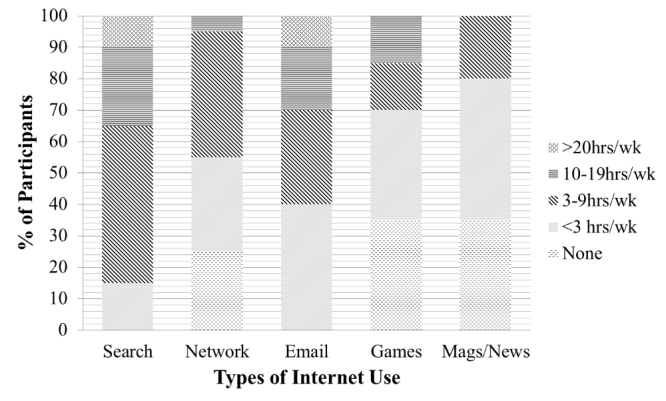
Type and amount of Internet use.

Of the 42 patients contacted, 20 (48%) agreed to participate in this feasibility study. Table 1 presents the sample characteristics. The average age of the sample was 53 years old (range, 39-72 years), with an average duration of diabetes of 12 years (range, 3 months to 25 years). Sixty-five percent (n=13) of the participants were white and 35% (n=7) were African American, 95% (n=19) were female, 55% (n=11) were married, and 25% (n=5) lived alone. In terms of socioeconomic status, 65% (13) had a bachelor’s degree or higher and 20% (n=4) had less than a college degree, 55% (n=11) were employed full or part-time, and 70% (n=14) had annual incomes ≥ $50,000. Ninety percent (n=18) of the participants reported having a family history of diabetes, 25% (n=5) were using insulin, 45% (n=9) were on oral diabetes medications, 25% (n=5) were on both oral medications and insulin, and 70% (n=14) reported attending at least one diabetes class in the past. All participants reported spending 3 or more hours per week on the Internet, 75% reported spending greater than 3 hours/week using social networks such as Facebook, 60% reported spending greater than 3 hours/week emailing, 30% reported playing Internet games greater than 3 hours/week, and 85% reported searching online greater than 3 hours/week. See Figure 2 for types and amount of Internet use.

**Table 1:** Sample characteristics of participants in SLIDES (N=20).

Attribute		n (%)
**Gender**		
	Female	19 (95)
	Male	1 (5)
**Age (yr)**		
	<45	3 (15)
	45-54	7 (35)
	55-64	6 (30)
	65-74	3 (15)
	Missing	1 (5)
**Race**		
	White	13 (65)
	African American/black	7 (35)
**Marital status**		
	Single	7 (35)
	Married	11 (55)
	Divorced	2 (10)
**Living with**		
	Spouse/Partner	11 (55)
	Children	4 (20)
	Other relatives	2 (10)
	Other	2 (10)
	None of the above	5 (25)
**Education**		
	Technical/trade school	2 (10)
	Some college	2 (10)
	Associates degree	3 (15)
	Bachelor’s degree	4 (20)
	Master’s degree	9 (45)
**Employment**		
	Full-time	7 (35)
	Part-time	4 (20)
	Retired	4 (20)
	Not employed	5 (25)
**Income level (US $)**		
	25,000-34,999	4 (20)
	35,000-49,999	2 (10)
	≥50,000	14 (70)
**Family history of diabetes**		
	Mother	9 (45)
	Father	12 (60)
	Sister(s)	5 (25)
	Brother(s)	6 (30)
	Children	2 (10)
**Medications**		
	On oral medications	14 (70)
	On insulin	10 (50)
	On other medications	2 (10)
**Ever attended diabetes support group**		14 (70)

### Participation Rates and Activity

**Figure 3:**
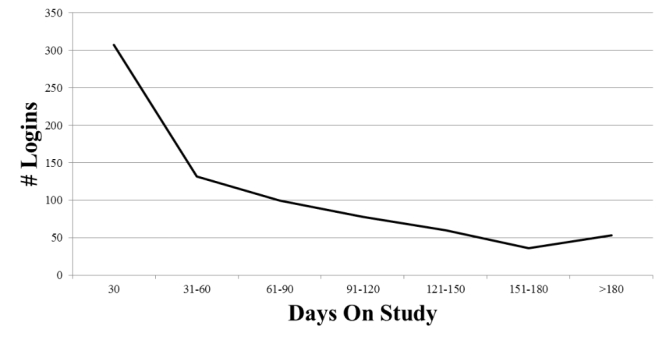
Number of log-ins into the SLIDES site.

Participants logged into SLIDES a total of 766 times with a mean of 38 times, a median of 35 times, and a range of 1-113 times per participant over the 6 months they were in the study. Seventy-five percent of the log-ins occurred in the first 3 months with the majority in the first month (Figure 3). Participants logged in for an average of 43 minutes per session with a median of 33 minutes per session. A subgroup of participants (n=14, 70%) were very active during the first 3 months in the study. This 3-month period represented one of the 12-week DSM class periods held in the site over the 6-month study period. These participants logged into the site a total of 712 times with a mean of 51 times, a median of 42 times, and a range of 19-113 times. On average, they logged into the site for 40 minutes per session. Most log-ins occurred around the times of scheduled classes or support sessions in SLIDES; however, some participants logged in on their own on other dates or times and interacted with the resources in the site (ie, grocery items, books, etc). In terms of communication, participants used voice communication a majority of the time when in the VE with others; text communication was only used during technical Second Life issues with voice functionality.

**Figure 4:**
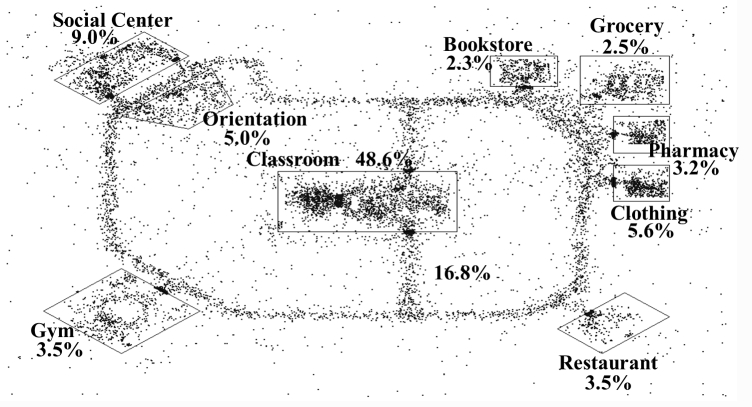
Heat map of locations visited.

The participants in this study visited every location in the VE. However, they spent the majority of their time in the classroom (48.6%) because DSMT classes were held there twice weekly. The second most frequently visited area was the “outdoors” area on the site. As shown in the heat map (Figure 4), we discovered from our focus group that some of the participants used the outside area as a way to reduce stress because they could hear birds chirping or water in the background. The third most frequent area where the participants spent time was the social center (9.0%), a place they visited to socialize with other participants. Interestingly, the fourth most frequented area was the clothing store (5.6%), which allowed participants to individualize their avatars with clothing, hair, and general appearance.

A total of 297 of 394 (75%) objects such as food items, books, menus, websites, videos, and pharmacy items were “handled” by 19 of the participants while in SLIDES. Participants interacted with these 297 objects a total of 1180 times. Participant interaction with specific types of objects within these locations is delineated in Table 2.

### Perceived Usefulness and Ease of Use

Overall, participants anticipated SLIDES to be useful at baseline (mean 2.14 SD 0.73) and this continued at 3 months (mean 2.02 SD 1.21) and at 6 months toward extremely likely (mean 1.68, SD 0.79). Although there was a trend toward extremely useful, there was not a statistically significant improvement (*P*=.07) between baseline and 6 months. With regard to ease of use, participants ranked SLIDES at baseline, 3, and 6 months between slightly and quite easy to use (baseline mean 2.48 SD 0.87; 3-month mean 2.73, SD 1.34; 6-month mean 2.33, SD 1.16). There were no statistically significant differences over time in ease of use; and baseline anticipations matched experiences during the study participation.

**Table 2:** Summary of all object interactions by participants.

Location of objects	Object category (items in store/items examined)	Object subcategory (number of interactions, %)	No. of participants who interacted with objects in location	Total no. of times object was interacted with by all participants	Number of interactions with objects per participant, mean (SD)
Grocery store	All food items (146/211)		19	408	22.66 (29.79)
		Meat, poultry, fish, nuts, beans (90, 22%)			
		Beverages (82, 20%)^a^			
		Fats, oils, sweets (61, 15%)^a^			
		Cereal/breads (33, 8%)^a^			
		Dairy (33, 8%)^a^			
		Vegetables (33, 8%)^a^			
		Frozen foods (33, 8%)^a^			
		Snack foods (20, 5%)^a^			
		Fruit (16, 4%)^a^			
		Rice/pasta (8, 2%)^a^			
Bookstore	Books (20/22)		15	63	4.2 (3.74)
		Nutrition (56, 89%)^a^			
		Diabetes management (4, 6%)^a^			
		Medication/treatment (3, 5%)^a^			
	Websites (self-care) (11/18)		15	105	7.0 (6.15)
Restaurant	Menus (56/64)		16	192	12 (18.46)
		Chain eat-in restaurants (106, 55%)^a^			
		Chain drive-through restaurants (86, 45%)^a^			
Pharmacy	All items (49/63)		15	104	6.93 (5.67)
		Blood glucose monitors (42, 40%)^a^			
		Dental, foot, skin care (25, 24%)^a^			
		Injection supplies (15, 14%)^a^			
		Mobility products, scales, diabetes specialty supplements (23, 22%)^a^			
	Medications (Rx) (10/11)^a^				
		Lipid lowering (21, 20%)			
		Oral antihyperglycemic (66, 63%)			
		Insulin (18, 17%)			
Gym	Exercise videos (cardio, yoga, strength training) (3/3)		17	134	7.88 (9.31)
Community center	Recorded classes (11/12)		16	73	6.63 (4.05)
		Intro to diabetes (15, 21%)^a^			
		Healthy eating (10, 14%)^a^			
		Exercise (10, 14%)^a^			
	Forum		12	101	8.41 (10.1)

^a^Items reviewed most often.

### Focus Groups

Eight of the participants attended the focus group. Six of these focus group participants were active users of the SLIDES VE and 2 were much less active participants. Four categories were discussed based on the focus group questions: expectations of the program, positive aspects of SLIDES, negative aspects of SLIDES, and suggestions for changes. The themes noted in the data coding were consistent with the theoretical framework (environmental factors, personal factors, and behavior) for the study approach, and are summarized in Table 3. The environmental factors were addressed in the participant comments through the themes of informational and community resources, social interaction, physical realism, and aspects of the usability of the site. The personal factors were addressed in the participant comments through the theme of diabetes knowledge, and behavior was addressed through self-management behavior change.

The focus group confirmed existing problems that we were aware of with the site and offered new insights for improvement of the site in future versions. With regard to the user expectations, all users thought that site provided resources that improved their knowledge of diabetes. The majority of the comments regarding the positive aspects of the site revolved around the informational resources and social interaction. The participants thought that the site provided a source of new information not only in the various locations in the site such as the grocery store, restaurant, and bookstore, but they also liked the interactive nature and informational aspects of the weekly classes. One participant stated that she learned something new every week. The majority of the positive comments on social interaction addressed their ability to interact with others synchronously and hearing about the experiences of others and learning through other participants “stories” about their disease. The majority of the comments about the negative aspects of the site were categorized as usability problems. For example, participants found the background noises made by other participants chewing food or just general home sounds, such as people talking or dogs barking, very annoying. Because the majority of conversations were synchronous, if the users did not mute their microphones, all of the sounds within their respective homes were heard by all participants and thus distracting. This required the moderators of social events such as the classes to ask participants to mute their individual microphones until they were ready to speak. Other problems identified by the participants had to do with the complex functionality of Second Life. These problems included navigational issues such as moving their avatar from one location to another, having difficulty with sound, and general computer problems that did not meet the specifications required by Second Life. Additionally, 1 participant who thought that there would be more participants in the site was referring to an age diversity issue. She was hoping for more people in her age group. Finally, the suggestions for future changes provided us with ideas on how to improve the site in the future. The majority of these suggestions were about how to improve social interaction such as including tasks to complete together as group such as homework, scavenger hunts, and in general group bonding activities. The participants thought that these types of activities would have helped them to bond earlier during their time in the site.

**Table 3:** Focus group results.

Categories	Themes (n, %)	Examples
**Expectations (N=10)**		
	Informational resources (3, 30%)	“The educational material was a plus too”
	Met expectations (2, 20%)	“Met expectations in terms of discussion”
	Diabetes knowledge (2, 20%)	“Learn a few things”
	No expectations (2, 20%)	“I had no expectations and was delighted”
	Social interactions (1, 10%)	“Intrigued by being with other people with diabetes”
**Positive aspects of the site** **(N=55)**		
	Informational resources (17, 31%)	"Liked the comments on the items in the grocery store"
	Social interaction (16, 29%)	“I did enjoy interacting with others”
	Diabetes knowledge (6, 11%)	“have had diabetes for 25 years, but learning new things”
	Usability (6, 11%)	“I am a click and find person—like things immediately at my disposal”
	Community resources (5, 9%)	“Liked the gym, helped me to exercise”
	Physical realism (3, 5%)	“Liked the seasons changing in the site”
	Self-management behavior change (2, 4%)	“Literally changed my life in terms of treatment with insulin—rarely now takes insulin during the day”
**Negative aspects of the site (N=34)**		
	Usability (26, 76%)	“background noises from others – home sounds, chewing”
	Social interactions (3, 9%)	“Expected larger group of participants”
	Informational resources (3, 9%)	“Need clarity on nutrition information by serving”
	Behavioral realism (2, 6%)	“Avoided gym ‘just like in real life’”
**Suggestions for changes to site (N=31)**		
	Social interaction (13, 42%)	“Would be good to have group exercises”
	Informational resources (9, 29%)	“More variety in the grocery store”
	Usability (5, 16%)	“Would like to hear (bots)and read feedback”
	Diabetes knowledge (3, 10%)	“Would like nutritionist or other specialists at classes (podiatry, pharmacist)
	Community resources (1, 3%)	“Would like a walking path”

### Physiological Outcomes

There were no changes noted from baseline to 6 months in systolic or diastolic blood pressure; however, overall the participants had good blood pressure control (Table 4). The changes that were noted were in weight and HbA1c. Although there was not a statistically significant change in either of these indicators, there was an average weight loss of 9.1 lb from baseline to 6 months. HbA1c also decreased from 7.51% (SD 1.15%) at baseline to 6.92% (SD 1.37%) at 6 months.

**Table 4:** Physiological and psychosocial outcomes.

Variable		Baseline (N=18) Mean (SD)	3 Months (N=14) Mean (SD)	Change at 3 months	6 Months (N=13) Mean (SD)	Change at 6 months
HbA1c (%)		7.51 (1.15)	7.14 (1.24)	*t*_13_=0.54; *P*=.60	6.92 (1.37)	*t*_11_=0.20; *P*=.85
Weight (lb)		217.5 (45.2)	215.7 (45.8)	*t*_.61_=0.61; *P*=.55	208.4 (43.9)	*t*_12_=1.04; *P*=.32
BMI (kg/m^2^)		37.4 (7.9)	37.2 (8.3)	*t*_14_=0.56; *P*=.58	36.2 (8.5)	*t*_12_=1.14; *P*=.28
Systolic blood pressure (mm Hg)		131.3 (13.0)	129.6 (14.5)	*t*_14_=0.47; *P*=.64	130.1 (10.5)	*t*_12_=−0.38; *P*=.71
Diastolic blood pressure (mm Hg)		74.8 (10.8)	74.7 (11.2)	*t*_14_=0.31; *P*=.76	78.1 (9.4)	*t*_12_=−0.91; *P=*.38
Self-efficacy (score scale 1-5)		3.89 (0.81)	4.45 (0.67)	*t*_13_=−2.3; *P*=.036	4.64 (0.39)	*t*_11_=−2.73; *P*=.02
Social support (score scale 1-7)		4.61 (1.25)	5.45 (1.07)	*t*_13_=−2.1; *P*=.056	6.35 (0.44)	*t*_11_=−4.0; *P*=.002^a^
Diabetes knowledge (% score)		89.1 (4.04)	93.9 (5.25)	*t*_13_=−1.73; *P*=.108	88.2 (15.0)	*t*_10_=0.29; *P*=.77
**Self-management (days per week)**						
	Dietary	4.13 (1.42)	4.5 (1.67)	*t*_13_=0.51; *P*=.618	4.75 (1.45)	*t*_11_=−0.69; *P*=.50
	Exercise	3.07 (2.03)	2.43 (1.74)	*t*_11_=0.00; *P*=1.00	2.79 (2.26)	*t*_9_=−0.76; *P*=.46
	Blood sugar testing	5.15 (2.04)	4.79 (2.08)	*t*_13_=0.18; *P*=.859	4.83 (2.28)	*t*_11_=0.70; *P*=.50
	Foot care	3.68 (2.08)	4.61 (2.19)	*t*_13_=−1.3; *P*=.213	6.17 (1.54)	*t*_11_=−2.54; *P*=.03^a^

^a^Significant difference at *P*>.05 level.

### Psychosocial and Behavioral Outcomes

Table 4 presents a summary of the effect of SLIDES on psychosocial diabetes outcomes including self-efficacy, social support, self-management behaviors, and diabetes knowledge. Participants were fairly knowledgeable about diabetes at baseline with a mean score of 89.1 (SD 4.04). There were no significant changes in knowledge level from baseline to 6 months post-intervention. At baseline, self-efficacy was neutral to moderate (mean 3.89, SD 0.81); however, this improved to moderate at 3 months (mean 4.45, SD 0.67) and then improved significantly toward high (*P*=.02) at 6 months (mean 4.64, SD 0.39). Similarly, participants ranked their social support for help in managing their diabetes between neutral and moderate (mean 4.61, SD 1.25) at baseline. However, their perception of social support changed at 3 months to moderate (mean 5.45, SD 1.07) and significantly (*P*=.002) increased at 6 months (mean 6.35, SD 0.44). For self-management behaviors, participants only showed statistically significant improvement in the number of days per week performing foot care (mean 3.68 SD 2.08 days per week to mean 6.17, SD 1.54 days per week at 6 months). No significant changes were noted in diet, exercise, or blood sugar testing. No participants reported smoking.

### Presence

Participant perception of presence [45] (the feeling of “being there”) changed slightly from 3 months to 6 months. At 3 months, the mean response was somewhat or 4.44 (SD 0.73; mean range, 2.74-5.59) on a 7-point Likert scale. At 6 months, the mean response did not significantly change, but increased slightly to 4.64 (SD 46; mean range, 4.04-5.56). We examined the factors of control, sensory, distraction, and realism within the presence questionnaire, but found no significant differences between the overall mean and the individual factors.

### Copresence

Copresence (the sense of being with other virtual humans) changed slightly from 3 months to 6 months. At 3 months, the mean score on a 7-point Likert scale for copresence was (slightly agree) 5.25 (SD 1.42) and at 6 months, the score was not significantly different (toward agree) at 5.78 (SD 1.35). Although a higher score indicates a greater level of copresence, and the participants average score did increase from 3 months to 6 months, there was not a statistically significance change. Embarrassment and likability are two other constructs to measure social response to others in a VE [47]. For embarrassment (degree of social influence), the mean score at 3 months was (slightly disagree) 3.44 (SD 2.08) and at 6 months, the score was (toward neutral) 3.99 (SD 2.03). Again, although a higher score shows a greater readiness to carry out embarrassing acts in front of others, there was no statistically significant difference between 3 and 6 months. These scores correlate well with how the participants acted while in the site. For example, when the participants lost their clothes while changing the clothes on their avatars, they were so embarrassed that they immediately left the site and the study coordinator had to intervene and redress their respective avatars. Likability (social response to others) was also measured into a single score at both 3 and 6 months. At 3 months, the composite score was (slightly agree) 5.4 (SD 1.23) and at 6 months, the score was (agree) 6.06 (SD 0.76); however, there was not a statistically significant difference between time points.

## Discussion

### Principal Results

This study examined the feasibility and acceptability of an eHealth program utilizing a digital VE platform that provided diabetes education and self-management support. In addition, we examined physiological outcomes (HbA1c levels, BP, BMI) and psychosocial outcomes or mediators (diabetes knowledge, self-management behaviors, self-efficacy, perceived support, presence, and copresence) to determine preliminary effects of participation in the VE intervention. To our knowledge, this is the first study to explore the feasibility of VEs as a medium to improve T2D self-management in patients. This study showed that not only was the VE easy to use even for our elderly participants (age >65), but the participants overall found the environment and the synchronous interaction with peers and educators useful.

Participants were very engaged in the VE particularly within the first 3 months or the total time of one diabetes class series (12 weeks). Once they completed the class series, they continued to come into the site, but not as frequently. There were two possible reasons for this: (1) the class series was completed, and (2) they had reviewed all informational resources on the site. A subgroup of 6 participants who had formed a supportive social group continued to return to the site to attend weekly group sessions. This ongoing group demonstrated that the classes and resources were important, but social support was very significant in their disease management, as we know from the literature to date [51]. Two factors may have facilitated this bonding between the participants; presence (the feeling of being there), copresence (the sense of being there with others), and the ability to communicate synchronously with other participants in real-time. The sense of presence is dependent upon immersion and interaction. Because our participants interacted synchronously with each other via voice, their virtual experience was more realistic and thus the greater sense of bonding among these participants. Additionally, the sense of presence and copresence prompted the participants to relate personal diabetes experiences and thus promoted a social learning situation. Social learning is based on the premise that understanding of content is socially constructed through conversations and interactions with others [52]. In addition to these potential reasons for drop off in utilization and persistent use by a subgroup of participants, we will conduct further analysis in our data visualization and case-based work, which is ongoing. This will allow us to address potential subgroup efficacy and participation characteristics as described by Eysenbach in VE and other Internet-based intervention research [53]. Although a cost analysis of this intervention was not within the scope of this study, sustainability of an intervention such as this one, we believe is directly linked to participant engagement. Keeping participants engaged requires not only a dynamic interface with sustained changes to the content, but also a supportive social group where individuals have the potential to bond over a period of time.

Participants spent the majority of their time in the classroom (48.6%), followed by the outside areas of the VE (16.8%), and the Social Center (9.0%). It was expected that the majority of participant time would be spent in the classroom because that is where they attended class once or twice a week. However, it was surprising to find that a significant amount of time was spent outside. The time spent moving from one location in the VE to another (see path around site on heat map Figure 3) was expected for transit. The activity on the “grassy areas” of the VE were unexpected, but clarity was obtained from our focus group, when we learned that participants used the site to help reduce anxiety or stress by sitting in the open outdoor areas and listening to the ambient sounds such as water lapping at the shore, birds chirping, or wind blowing. Virtual environments that are immersive provide both visual and auditory information [54]. This sensory information changes as the avatars move from place to place within the environment similar to real life, thus promoting a sense of presence or actually being there [54].

Participants also spent a significant amount of time interacting with objects. The top objects interacted with were food items in the grocery store. This is not surprising as people with diabetes find dietary issues and guidelines to be the most challenging [55]. Considering that social cognitive theory includes knowledge application, we embedded information in the virtual objects (user had to click on the object to see the information) such as nutritional feedback and suggestions for change in unhealthy food choices in each item in the grocery store. These external pieces of information served as cues to action to trigger good decision-making processes in terms of food choices. Participants in the focus group reported using this information to make healthier eating choices.

Statistically significant improvements were found in 3 behavioral and psychosocial outcomes at 6 months: social support, self-efficacy, and foot care. This was not expected given the small pilot sample size. However, we interpret these with caution given the sample size and heterogeneity in terms of baseline demographics and metabolic control. Although we did not find statistical significance in the physiological indicators, we did find some weight loss, and associated decrease in BMI. The mean weight loss of 9.1 lb is clinically relevant and we will need to explore case-based analysis further regarding improvements among subgroups by participation and behavioral outcomes characteristics. Interestingly, this weight loss occurred in the absence of significant dietary and physical activity changes, which could also indicate inaccuracy or variation in weight measurement in the clinic settings and needs to be interpreted with caution. Attaining higher quality and consistently collected measures are being studied in our current larger trial. HbA1c decreased from baseline to 6 months post-intervention, reaching a level of less than 7% at 6 months, although this is interpreted in the context that metabolic control was relatively good at baseline. It is thought that interactive health interventions exert their effects by a combination of enhanced self-efficacy and knowledge, enabling patients to change their health behaviors, leading in turn to changes in clinical outcomes [19]. Therefore, these findings are promising because significant improvements in self-efficacy and support may affect DSM and metabolic control in larger trials of VEs.

### Limitations

Our findings are limited by the small pilot study sample and lack of comparison or control group, and will need to be tested in our future larger randomized controlled trial. This study primarily focused on feasibility and usability of VEs in the context of DSM and support. Although the study sample was diverse in terms of age and number of years with diabetes, it was primarily well-educated women with at least moderate income levels. We need to demonstrate the efficacy in a larger, more diverse sample. Broadening the locations and demographics of targeted participant recruitment in our larger trial, and ensuring that a VE is developed that appeals across age, race/ethnicity, and gender groups is a primary focus. We will also need to address the issue of enrolling a sample of activated participants who start with fairly good metabolic control in our future efficacy studies, and determine the ability to engage and improve outcomes among those with poor metabolic control who are less likely to participate in DSME in general. In terms of results, the reliance on clinical measures (weight, BP, HbA1c) from medical records due to the financial constraints of a pilot study resulted in some missing data and possible inconsistency in collection of these measures within the clinic setting. Therefore, the clinical outcomes must be interpreted with caution. Finally, participation within the site dropped after 12 weeks. We believe this was problematic because we did not keep the site content dynamic [56]. Our further study in this area will address these issues and attempt to explore participation and engagement over time.

### Conclusions

e-Health applications such as SLIDES enable patients with chronic diseases such as diabetes to become more engaged with the self-management of their disease. Evidence shows that people who use e-Health resources have better social support for their disease, increased knowledge, and gains in self-efficacy [57]. This study clearly showed a difference in social support and self-efficacy after 6 months of participation that substantiates these previous findings. Because little is known about usability, acceptability, and efficacy of health interventions in a VE, this study constituted an important, innovative first step in exploring the potential of VEs in facilitating DSM. Using constructs from social cognitive theory and constructs from a VE theoretical framework in the development of this virtual community, we showed that developing environments such as these have the potential to motivate and support people to effectively self-manage their type 2 diabetes. The preliminary data demonstrated that VEs provide a feasible, useful, and effective platform for patients and educators. Flexible access to both synchronous and asynchronous diabetes education, skill building activities, and support from a home computer removed barriers to attending traditional clinic-based meetings. This program has potential for improving DSM in an easily disseminated alternative model on the Internet that may promote more effective resource utilization by reaching increased numbers of participants across geographic boundaries and potentially decreasing institutional diabetes education resources while maintaining the sense of personal interaction that has been shown to be important in successful diabetes self-management interventions.

## Abbreviations

BMI: body mass index
BP: blood pressure
DES-SF: diabetes empowerment scale-short form
DSM: diabetes self-management
DSMT: diabetes self-management training
HbA1c: glycosylated hemoglobin
SLIDES: second life impacts diabetes education & self-management
T2D: type 2 diabetes
VE: virtual environment
